# Study on Effects of Cyclophosphamide Combined with Vinorelbine in Advanced Small Cell Lung Cancer and Anteroposterior Changes in MRI

**DOI:** 10.1155/2022/3104879

**Published:** 2022-08-08

**Authors:** Zhichun Li, Liliang Ren

**Affiliations:** ^1^Pharmacy, Shandong Haiyang Traditional Chinese Medicine Hospital, Yantai 265100, Shandong, China; ^2^Department of Imaging, Yantai Mountain Hospital, Yantai 264001, Shandong, China

## Abstract

**Objective:**

To explore the effects of cyclophosphamide combined with vinorelbine in advanced small cell lung cancer (SCLC) and anteroposterior changes in MRI.

**Methods:**

The clinical data of 90 patients with advanced SCLC admitted to our hospital from April 2020 to April 2021 were retrospectively analyzed. They were divided into the control group and the study group according to the order of admission, with 45 cases in each group. The control group received the routine treatment, while the study group was treated with cyclophosphamide and vinorelbine to compare the indexes of imaging data and clinical indicators between the two groups before and after treatment.

**Results:**

There was no significant difference in the indexes of imaging data between the two groups before treatment (*P* > 0.05), and the indexes of imaging data in the study group were visibly lower than those in the control group after treatment (*P* < 0.001). The DCR in the study group was significantly higher than that in the control group after treatment (*P* < 0.05), while the QLQ-C30 scores and serum indices of the study group after treatment were significantly lower than those of the control group (*P* < 0.001).

**Conclusion:**

Patients with advanced SCLC were treated with cyclophosphamide and vinorelbine, which can effectively improve the quality of life and reduce the expression of inflammatory factors. This treatment model has a higher application value, and the treatment value is also reflected compared with the routine treatment. At the same time, the permeability parameters obtained by MRI can predict the therapeutic effects of cyclophosphamide and vinorelbine, and further studies are helpful to establish a better solution for patients.

## 1. Introduction

Lung cancer is divided into small cell lung cancer (SCLC) and nonsmall cell lung cancer (NSCLC), and SCLC accounts for about 15%–20% [1]. Based on the statistical data from the World Health Organization (WHO) [2], the morbidity and mortality of lung cancer are high, with about 1.8 million new cases worldwide each year, accounting for 13% of all tumors, and lung cancer has the highest morbidity and mortality of malignant tumors in both urban and rural areas in China [3]. The above data are warning that lung cancer poses a serious threat to the lives of patients. Characterized by low differentiation, rapid growth, long doubling time, and extensive metastasis, SCLC is one of the tumors with the highest malignant degree in lung cancer, which can produce a variety of active hormones and neuroendocrine markers [4, 5]. Most patients have distant metastasis at the first diagnosis, which is generally not suitable for surgical treatment; although SCLC is sensitive to radiotherapy and chemotherapy, the drug resistance occurs soon, thus leading to the disease progression [6, 7]. SCLC has fewer treatment methods compared with NSCLC, so seeking a suitable treatment has become a hot topic for cancer scholars.

Cyclophosphamide, a widely used anticancer drug at present, belongs to the cell cycle nonspecific agents, whose effects are confirmed in chronic lymphocytic leukemia, malignant lymphoma, nasopharyngeal cancer, breast cancer, and multiple myeloma [8]. Vinorelbine, as an antitumor drug of vinca alkaloids inhibiting cell division, can effectively inhibit the expansion of physical cancer cells, and the effects have been confirmed in advanced ovarian cancer and metastatic breast cancer [9]. Clinical observation is often combined with imaging methods to better monitor the therapeutic effects of cyclophosphamide and vinorelbine. In recent years, MRI has been widely used in pulmonary diseases, and high-quality lung images with sufficient spatial resolution can be generated by optimizing the scanning series. Kudoh et al. [10] have pointed out that MRI technology has obvious advantages in evaluating the therapeutic effect of SCLC. In addition, MRI can comprehensively detect the changes in the cellular structure of tumor mass, blood flow changes, and vascular properties within the tumor before and after treatment and evaluate the therapeutic effect of solid cancer. At the same time, MRI as a noninvasive imaging technology for predicting therapeutic response can not only effectively evaluate the permeability of tumor microvasculature but also evaluate the effects of antiangiogenic drugs on tumor microvasculature, to provide a certain reference for clinical treatment. However, as a retrospective study, the definition of symptoms or diseases may change, and the study conclusions are also susceptible to selection bias and recall bias. To further explore the effects of cyclophosphamide combined with vinorelbine in advanced SCLC and anteroposterior changes in MRI, this study adopted this scheme and carried out the joint clinical intervention on subjects to provide more clinical evidence-based proof for such patients.

## 2. Materials and Methods

### 2.1. General Information

The clinical data of 90 patients with advanced SCLC admitted to our hospital from April 2020 to April 2021 were retrospectively analyzed. They were divided into the control group and study group according to the order of admission, with 45 cases in each group. This study was consistent with the Declaration of the World Medical Association [11].

### 2.2. Recruitment of Study Subjects

Inclusion criteria: ① patients were in line with the diagnostic criteria in the diagnosis and treatment guidelines of small cell lung cancer [12] and were diagnosed with advanced SCLC by histological or cytological pathological tests. ② Patients had lesions that could be evaluated by imaging. ③ Patients' expected survival period exceeded 3 months. ④ Patients had no obvious organ dysfunction syndrome or another tumor. ⑤ Patients had a clear tumor boundary. ⑥ The ECOG scores were less than 2 points.

Exclusion criteria: ① patients with resistant hypertension and active hemorrhage. ② Patients with severe diseases in the heart, brain, liver, and kidney and patients who had the transfer of symptoms. ③ Patients with claustrophobia or other reasons that could not cooperate with MRI examination. ④ Patients with the mental illness. ⑤ Patients with the metal implant in their body. ⑥ Patients who were easy to have the intrapulmonary or distant metastasis.

### 2.3. Methods

#### 2.3.1. Control Group

Patients in the control group were treated with 850 mg of apatinib (manufacturer: Jiangsu Hengrui Medicine Co., Ltd.; NMPA approval No. H20140103; specification: 0.25 g) by oral administration at 30 min after the meal was delivered in the warm water once a day, with the treatment time of 3 months.

#### 2.3.2. Study Group

According to the relevant data [13], patients in the study group received cyclophosphamide injection (manufacturer: Hanhui Pharmaceutical Co., Ltd.; NMPA approval No. H20093392; specification: 0.2 g) by single-drug intravenous injection at a dose of 500 mg/m^2^/time–1000 mg/m^2^/time according to body surface area. 20 ml–30 ml of saline was added once a week for consecutive 2 times, and the blood routine of patients was monitored regularly during the medication, with “with the adjustment of medical dosage when the number of leukocytes was reduced. The treatment was repeated after 1-week or 2-week rest. At the same time, vinorelbine (manufacturer: China Meheco Kangli Pharma Co., Ltd.; NMPA approval No. H20040477; specification: 10 mg) was dissolved in 1 ml of injection water before use, and then 0.9% of sodium chloride injection at a dose of 10 ml was added for dilution, with the first dosage as 2.5 mg and the largest dosage as 5 mg at the injection rate of 1 mg/min-2 mg/min, while the total amount could not exceed 10 mg–15 mg. The incidence of myelosuppression in patients was monitored before and after treatment, and the inspection of blood cell count was performed before medication each time, with a treatment time of 3 months.

#### 2.3.3. MRI Inspection

After the patients had dorsal position, the MRI (manufacturer: Marconi Medical Systems, Inc.; specification: Infinion 1.5 T) inspection was performed using the 8-channel phased-array surface coil. When the patients were in a calm state without effect on respiration, the abdominal belt was tightened to make the anterior chest close to the coil, and the patients were ordered to adopt the abdominal respiration. The fasting for 6 h was performed before inspection, and the chest ornaments, neck ornaments, and other metal objects were removed to reduce the respiratory motion artifact, with the scanning from the thoracic entrance to the pulmonary lower bound. The setting parameters were as follows. In T1WI, TR was 250 ms–550 ms and TE was 1–15 ms. In T2WI, TR was 1500–7500 ms and TE was 45–75 ms. In coronal T2WI, TR was 1500–7500 ms and TE was 75–145 ms. In axial DWI, TR was 1500–7500 ms and TE was 75–145 ms. The slice thickness was 5 mm, layer spacing was 2 mm, the matrix was 225*∗*245, F0V was 35*∗*35, the number of excitation (NEX) was 4, and diffusion sensitive gradient *b* factor was 0 and 750 s/mm^2^.

#### 2.3.4. Image Processing Analysis

All data were performed by GEN IQ postprocessing software at the GE adw4.7 workstation, and the data were collected by two radiologists to determine whether the images could be measured and calculated. The standard was that the images had no change and did not affect the false shadow of data measurement.

### 2.4. Observation Indices

The indexes of image data in the two groups before and after treatment were observed, and the interesting region was manually drawn on the cross-section with the maximum diameter after excluding the visible blood vessels and necrotic tumors. The software package was used to analyze the volume transfer constant (Ktrans), extravascular extracellular volume fraction (Ve), and rate constant (Kep).

The clinical efficacy of the two groups after treatment was evaluated according to the response evaluation criteria in solid tumors, RECIST [14]. If all target lesions in patients disappeared, and the duration exceeded 4 weeks, the disease was CR (complete remission). If the total length and diameter of the baseline lesion were reduced by ≥30% and the duration was ≥4 weeks, the disease was PR (partial remission). If the total length and diameter of the baseline lesion increased by ≥20% or a new lesion appeared, the patients were in PD (progressive stage). If the total length and diameter of the baseline lesion in the patients decreased but did not reach PR or increased but did not reach PD, the patients were in SD (stable stage). The disease control rate (DCR) = (CR + PR + SD)/total cases.

The European Organization for Research and Treatment of Cancer Core Quality of Life Questionnaire (EORTC QLQ-C30) [15] was used to evaluate patients' quality of life in the two groups after treatment. The scale was divided into four dimensions including 30 items, with a total of 126 points. The higher the score, the worse the quality of life.

The fasting venous blood (5 mL) of the two groups in patients after treatment was collected and centrifuged at 3000 r/min for 10 min to store the upper serum. The neuron-specific enolase (NSE) and cancer antigen 125 (CA125) in serum indices were detected using electrochemiluminescence assay, and carcinoembryonic antigen (CEA) was detected by chemiluminescence microparticle immunoassay. All operations were carried out according to the kit introduction provided by Wuhan Saipei Biotechnology Co., Ltd.

### 2.5. Statistical Treatment

The experimental data were analyzed and processed statistically by SPSS21.0, and the pictures were drawn by GraphPad Prism 7 (GraphPad Software, San Diego, USA). The enumeration data and measurement data were tested by *x*^2^ and *t*-test, indicated by [n (%)] and $\left(\bar{x}\pm s\right)$. When *p* < 0.05, the differences were considered to be statistically significant.

## 3. Results

### 3.1. Comparison of Baseline Data between the Two Groups

There was no significant difference in gender, age, BMI value, clinical stages, ECOG scores, educational level, occupation, religious belief, cases of family income, smoking, drinking, and place of residence between the two groups (*P* > 0.05). See details in Table 1.

### 3.2. Comparison of Indexes in Image Data between the Two Groups

There was no significant difference in the indexes of imaging data between the two groups before treatment (*P* > 0.05), and the indexes of imaging data in the study group were visibly lower than those in the control group after treatment (*P* < 0.001). See details in Table 2.

### 3.3. Comparison of DCR between the Two Groups after Treatment

The DCR in the study group was significantly higher than that in the control group after treatment (*P* < 0.05). See details in Table 3.

### 3.4. Comparison of QLQ-C30 Scores between the Two Groups after Treatment

**Figure 1:**
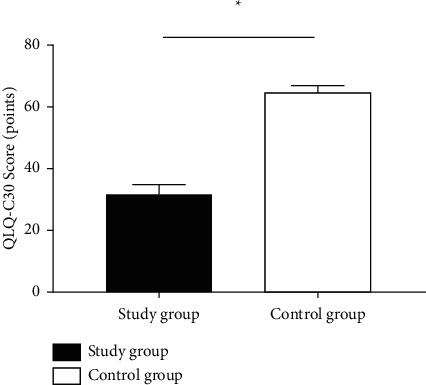
Comparison of QLQ-C30 scores between the two groups after treatment $\left(\bar{x}\pm s\right)$. *Notes.* The abscissa indicates the study group and control group, and the ordinate indicates the QLQ-C30 score (points). The QLQ-C30 scores of the study group and control group after treatment were (31.51 ± 3.38) points and (64.76 ± 2.00) points, respectively. ^*∗*^ represents a significant difference in QLQ-C30 scores between the two groups after treatment (*t* = 56.793, *P* < 0.001).

The QLQ-C30 scores of the study group after treatment were significantly lower than those of the control group (*P* < 0.001). See details in Figure 1.

### 3.5. Comparison of Serum Indices between the Two Groups after Treatment

The serum indices of the study group after treatment were significantly lower than those of the control group (*P* < 0.001). See details in Table 4.

## 4. Discussion

SCLC belongs to the neuroendocrine carcinoma, with the highest malignant degree in lung cancer, and the mortality is as high as 89% [15]. Characterized by low differentiation degree, strong neoplasm invasiveness, and rapid growth in SCLC, the metastasis can occur in the early stage of the disease, and most patients have developed into the middle or late stage at the time of diagnosis, thus missing the best treatment opportunity [16]. In addition, the medication of advanced SCLC is relatively expensive, which brings a heavy economic burden to the individual and family of patients and causes negative psychology such as depression and insufficient confidence in treatment, thereby further affecting the therapeutic effect. At the same time, most patients with advanced lung cancer lose the opportunity of thoracotomy surgery, with the operability of surgical resection less than 29%, but radiotherapy and chemotherapy are usually used in clinics in order to eliminate the tumor tissue [17]. In recent years, the new targeted therapy has been gradually improved and gets effective promotion. Therefore, it is necessary to select reasonable detection methods for early expectation and evaluation of curative effects, so as to timely and effectively adjust individualized treatment plans and prevent adverse risk accidents such as delay of disease caused by incorrect treatment and other factors [18]. The response evaluation criteria in solid tumors generally adopted in the clinical examination have the advantages of convenience and simplicity, but the evaluation has certain hysteresis due to the slow response of effect on the disease to therapeutic drugs. To predict the effects of drug therapy as soon as possible, it is particularly important to adopt reasonable detection methods to adjust the later treatment plan for the prognosis of patients. MRI is less involved in the evaluation criteria of solid tumors, but recent literature has gradually emphasized the inspection potential of this technique for tumor patients [19]. MRI scanning can not only reflect tumor function information such as microvascular density, formation of tumor vessels, and surface permeability of capillary but also provides morphological images for better analyzing the physiological structure of tissues and characteristics of functional metabolism. The results of this study could truly display the imaging indexes of the two groups before and after treatment, which also provided a basis for doctors to objectively evaluate the effectiveness of the treatment plan. At the same time, from the perspective of imaging indexes, the imaging indexes of the study group after treatment were significantly lower than those of the control group and before treatment (*P* < 0.001), confirming the application value of this treatment plan again. Kang-Lung et al. [20] have evaluated the short-term efficacy of lung cancer treatment by adopting the dynamic enhanced MRI scanning, and the parameters obtained by image processing were consistent with the results of this study, fully confirming that MRI parameters can be used as effective functions to provide the relevant basis for the therapeutic effect of new antitumor drugs. As a widely used antiangiogenic drug in clinics, apatinib can effectively block the transduction pathway of downstream signal and inhibit the generation of tyrosine kinase, thus achieving the purpose of curbing tumor growth. This drug mainly achieves the purpose of treatment by oral administration, and the treatment process can be completed without hospitalization, but the clinical efficacy of this treatment method has not reached the expectation, so it is difficult to meet the clinical needs. However, cyclophosphamide, as a kind of anticancer drug of alkylating agent widely used in clinical practice in recent years, will be decomposed into chloroethylamide through the catalysis of hepatic microsomal enzymes in patients, resulting in a strong effect of alkylation, which directly affects tumor cells and produces cytotoxicity effect [21]. Vinorelbine, a new type of semisynthetic vinca alkaloids, directly acts on the dynamic balance of tubulin and microtubule, inhibits the polymerization of tubulin, and causes the disintegration of microtubules in the division stage, and the cell mitosis of *G*2 and *M* phases are blocked leading to cell death in the interphase or anaphase [22]. Based on the pharmacological analysis and clinical treatment experience, this study found that cyclophosphamide was applied in the treatment of various malignant tumors, and vinorelbine has been approved as a single drug or combination drug with other medications for the treatment of NSCLC, while MRI with high resolution of soft tissue can explore the lung situation. Therefore, the solution in this study had high feasibility and could help patients better solve clinical problems. The combination of two drugs can effectively control the progression of advanced SCLC in patients. Shin-Ichi et al. [23] studied 41 patients with advanced SCLC who received the combination treatment of nivolumab and GP chemotherapy, and the DCR was 78.05% (32/41) after treatment, with a worse therapeutic effect than this regimen. The results of this study showed that the DCR of the study group after treatment was significantly higher than that of the control group (*P* < 0.05), indicating that cyclophosphamide combined with vinorelbine has a better therapeutic effect compared with routine treatment, which can effectively inhibit the development of disease, with benefits to the prognosis of patients.

At the same time, the results of this study pointed out that patients' quality of life in the study group was distinctly improved by the treatment of cyclophosphamide combined with vinorelbine, indicating that cyclophosphamide combined with vinorelbine is effective and could significantly bring clinical benefits. In addition, the relevant literature has pointed out that NSE, as a specific acidic protease in neurons and neuroendocrine cells, has a lower expression level in healthy people [23]. Chauhan et al. [24] have pointed out that about 69% of SCLC patients have a higher serum NSE level, and the changes in this index are related to the occurrence of multiple tumors such as medullary thyroid carcinoma, neuroblastoma, and melanoma. Kishikawa et al. [25] have found that CA125, as a glycoprotein involved in the protection of cell function and lubrication, is often found in normal tissues such as ovarian epithelium and thoracoabdominal membrane and has an abnormally high expression in the serum of endometrial cancer, cervical cancer, and other tumors, which is a common tumor marker. Some studies have found that CA125 also has an abnormal expression in patients with lung cancer, which is closely related to the efficacy of chemotherapy in patients with advanced SCLC. The index of CEA is lower in normal people, and its synthesis is more in tumor cells, which leads to the gradual increase of CEA levels in the blood of patients. The concentration of CEA in different stages of tumor is different, and its concentration in patients without metastasis is lower than that in patients with metastasis. The higher the tumor differentiation degree, the higher the concentration of CEA. In this study, the serum indices of NSE, CA125, and CEA were visibly decreased after treatment, suggesting that the above indices were negatively correlated with the therapeutic efficacy, which could be used as important indicators for monitoring the evaluation of therapeutic effect in advanced SCLC. The solution of this study also has many complexities. Since the study subjects were humans, this feature determines that the implementation process of this scheme will face humanistic, social, and ethical problems, so it is more complex and difficult than laboratory studies. The shortcomings of this study were as follows. First of all, the selected cases in this study were all patients in a local hospital, with a single source of cases. Secondly, the scales used for evaluation in this study had subjectivity and intentionality to a certain extent in the clinic when patients answered the questions so that the study design needs to be completed. Finally, there was a lack of long-term follow-up observation on the intervention effect of patients. Therefore, the study design should be improved in the future, the follow-up time should be extended, and the effects of cyclophosphamide combined with vinorelbine in advanced SCLC and the anteroposterior changes in MRI should be discussed in detail from multiple perspectives and aspects. Despite the improvement and progress of medical technology, there are still many difficulties in the treatment of advanced SCLC. Therefore, medical workers still need to continue to explore more efficient treatment and diagnosis models to provide a more reliable basis for the diagnosis and treatment of advanced SCLC. In summary, the preliminary conclusions of this study still need to be further improved.

## Figures and Tables

**Table 1:** Comparison of baseline data between the two groups.

Items	Study group (*n* = 45)	Control group (*n* = 45)	*x* ^2^/*t*	*P*
Gender			0.073	0.788
Male	37 (82.22%)	36 (80.00%)		
Female	8 (17.78%)	9 (20.00%)		

Age ($\bar{x}\pm s$, years)	60.69 ± 10.50	63.47 ± 10.05	1.283	0.203

BMI (kg/m^2^)	20.61 ± 0.57	20.61 ± 0.61	0.000	1.000

Clinical stages			0.049	0.824
Limited stage	29 (64.44%)	30 (66.67%)		
Extensive stage	16 (35.56%)	15 (33.33%)		

ECOG scores			0.104	0.748
0–1 point	40 (88.89%)	39 (86.67%)		
2 points	5 (11.11%)	6 (13.33%)		

Educational level			0.048	0.827
Senior high school and above	28 (62.22%)	29 (64.44%)		
Junior high school and below	17 (37.78%)	16 (35.56%)		

Occupation				
Civil servant	11 (24.44%)	10 (22.22%)	0.062	0.803
Teacher	10 (22.22%)	12 (26.67%)	0.241	0.624
Financial staff	11 (24.44%)	13 (28.89%)	0.227	0.634
Individual	7 (15.56%)	5 (11.11%)	0.385	0.535
Others	6 (13.33%)	5 (11.11%)	0.104	0.748

Religious belief			0.048	0.827
Yes	17 (37.79%)	16 (35.56%)		
No	28 (62.22%)	29 (64.44%)		

Cases of family income			0.124	0.725
≥3000 yuan/(months·person)	40 (88.89%)	41 (91.11%)		
<3000 yuan/(months·person)	5 (11.11%)	4 (8.89%)		

Smoking			0.051	0.822
Yes	30 (66.67%)	31 (68.89%)		
No	15 (33.33%)	14 (31.11%)		

Drinking			0.073	0.788
Yes	36 (80.00%)	37 (82.22%)		
No	9 (20.00%)	8 (17.78%)		

Place of residence			0.053	0.818
City	31 (68.89%)	32 (71.11%)		
Town	14 (31.11%)	13 (28.89%)		

**Table 2:** Comparison of indexes in image data between the two groups $\left(\bar{x}\pm s\right)$.

Groups	n	Ktrans		Ve		Kep	
		Before treatment	After treatment	Before treatment	After treatment	Before treatment	After treatment
Study group	45	976.54 ± 156.44	514.16 ± 79.19	765.94 ± 97.90	583.22 ± 62.84	2048.32 ± 439.66	906.65 ± 107.84
Control group	45	973.27 ± 147.13	744.17 ± 25.08	741.95 ± 50.77	629.61 ± 17.35	2145.10 ± 413.48	1394.14 ± 56.13
t		0.102	18.575	1.459	4.774	1.076	26.899
*P*		0.919	<0.001	0.148	<0.001	0.285	<0.001

**Table 3:** Comparison of DCR between the two groups after treatment [*n* (%)].

Groups	*n*	CR	PR	SD	PD	DCR
Study group	45	0 (0.00%)	27 (60.00%)	10 (22.22%)	8 (17.78%)	37 (82.22%)
Control group	45	0 (0.00%)	11 (24.44%)	9 (20.00%)	25 (55.56%)	20 (44.44%)
*x* ^2^						13.828
*P*						<0.001

**Table 4:** Comparison of serum indices between the two groups after treatment $\left(\bar{x}\pm s\right)$.

Groups	*n*	NSE (ng/ml)	CA125(U/ml)	CEA (ng/ml)
Study group	45	13.66 ± 0.88	46.41 ± 0.99	3.43 ± 0.30
Control group	45	16.97 ± 0.92	52.43 ± 0.85	5.93 ± 0.60
*t*		17.441	30.949	25.000
*P*		<0.001	<0.001	<0.001

## Data Availability

The data used to support the findings of this study are available from the corresponding author on reasonable request.
